# Role of Rebiopsy in Relapsed Non-Small Cell Lung Cancer for Directing Oncology Treatments

**DOI:** 10.1155/2015/809835

**Published:** 2015-01-29

**Authors:** Antti P. Jekunen

**Affiliations:** Clinical Cancer Research Center, Vaasa Oncology Clinic, Turku University, Hietalahdenkatu 2-4, 65100 Vaasa, Finland

## Abstract

*Background.* Currently, few rebiopsies are performed in relapses of advanced non-small cell lung cancer. They are not customary in clinical practice of lung cancer. However, it is not possible to properly target treatments in cases of relapse without knowing the nature of new lesions. *Design.* This paper comprehensively summarizes the available literature about rebiopsy and broadly discusses the importance of rebiopsy in advanced non-small cell lung cancer. *Results.* Altogether 560 abstracts were used as material for further analysis. 19 articles were about clinical rebiopsy in lung cancer and were reviewed in detailed manner. *Conclusions.* This review shows that rebiopsy is feasible in non-small cell lung cancer, and success rates can be high if rebiopsy is accompanied by adequate evaluation before biopsy. Its use may resolve the difficulties in sampling bias and detecting changes in cancer characteristics. In cases where treatment was selected based on tissue characteristics that then change, the treatment selection process must be repeated while considering new characteristics of the tumor. Rebiopsy may be used to predict therapeutic resistance and consequently redirect targeted therapies. Such knowledge may resolve the difficulties in sampling bias and also in selecting preexisting clones or formulating drug-resistant ones. Rebiopsy should be performed more often in non-small cell lung cancer.

## 1. Introduction

### 1.1. Imaging

Lung cancer is usually suspected in individuals who have an abnormal chest radiograph results or symptoms caused by either local or systemic tumor effects [1]. An initial diagnosis relies on imaging examinations when patients seek help for symptoms. Today, more tumor lesions are found secondarily in routine checkups. Chest X-ray and computer tomography (CT) scans are widely used. Positron emission tomography (PET) is a golden standard for staging of lung cancer. Additionally, it is used when doctors require more information about metabolic activity in certain lesions or when seeking lymph nodes or lesions for biopsy, in case of relapses and metastases.

### 1.2. Methods of Tumor Biopsy

In cases of peripheral tumor, ultrasound- or CT-guided percutaneous fine-needle aspiration or core biopsy is performed (Table 1). Video-assisted thoracoscopy (VATS) is used for wedge excisions and needle aspirations. A thoracotomy is usually an option when a lobectomy is being considered. Central tumors, often with symptoms such as repeated pneumonias and hemoptysis, can be diagnosed by sputum cytology. Bronchoscopy provides better samples with a brush, a fine-needle biopsy, and a core biopsy. Percutaneous-core needle biopsies, when it is possible to perform them, give larger samples of tissue material for further studies. However, a thoracotomy would be the best option when tissue sample size is important. Based on a recent meta-analysis, endobronchial ultrasound (EBUS) and electromagnetic navigation (EMN) bronchoscopy have the potential to increase the diagnostic yield of peripheral lung tumors [1]. A thoracoscopic biopsy of the pleura had the highest yield for diagnosing metastatic pleural effusion in a patient with lung cancer. When stereotactic high dose radiotherapy is considered tissue samples need to be taken before radiation, because afterwards there is nothing to be biopsied for. Acquiring adequate tissue samples for histological and molecular characterization of non-small cell lung cancer (NSCLC) is considered paramount.

Biopsy is used to characterize tumors. Here in this study, rebiopsy means biopsy after cancer progression on initial therapy and repeated biopsy is used for conditions where an initial biopsy was not adequate for diagnosis and a new biopsy is performed. Basic staining and immunohistochemistry are routine in pathological diagnosis and also useful in rebiopsy. Table 1 lists various means of obtaining tissue and gives estimation of tissue yields.

### 1.3. Risks of Biopsy

Taking sputum samples is without safety issues, while all others have some risk for complications. As needle size increases, risk level increases also for biopsy complications. Clearly, it is of importance to determine what risks are coming from the location of biopsy target. The most serious complications include pneumothorax and bleeding. Of course, in resections overall risk of general anesthesia needs to be calculated before operation.

### 1.4. Molecular Pathology

Molecular methods are becoming more common in the pathological diagnosis (Table 2). Molecular biology techniques, particularly gene-expression microarrays, proteomics, and next-generation sequencing, have recently been developed to facilitate molecular classification [2]. Proteomics can further characterize tissue with two-dimensional gels. Third-generation immunoassays and protein pathway circuit arrays are also being used experimentally. DNA is quite stable and can be genotyped by different oligonucleotide arrays, based on PCR or sequencing. RNA is more difficult to extract, as it is rapidly destroyed by ribonucleases if samples are not quickly frozen to −70°C after biopsy. RNA provides opportunities to measure gene expression by complementary DNA microarrays or microRNAs by sequencing. Many analyses are already part of a standard care (Table 2). Protein analysis by immunohistochemistry is routine and widely available. Gene testing is becoming a regular practice, and preparations for sending adequate tissue samples with sufficient numbers of malignant cells to central laboratories are becoming common practice in all clinical pathology laboratories. This process depends upon determining gene changes that are related to drug activity.

### 1.5. Changing Therapies on Genetic Mutations

Consequently, measurements are needed to direct therapies, thus justifying collecting biopsy samples. In NSCLC-type adenocancer, two mutations are widely used to direct treatments: an epidermal growth factor receptor- (EGFR-) activating mutation indicating use of gefitinib erlotinib and afatinib [3] and an ALK (anaplastic lymphoma kinase) gene rearrangement, indicating use of critsonitib [4, 5].

### 1.6. Mutations

In NSCLC, there are many variations and mutations in DNA, and it is only a matter of time and successful research before there are more predictive mutations available to clinical practice. The most frequent mutations in adenocarcinomas are in TP53, KRAS, and STK11 and EGFR genes. ALK mutations are measured in 3% to 5% of all lung adenocarcinomas. Genomic pathology provides an opportunity to stratify patients, based on genomic predictive features after successful rebiopsy, and consider changing treatment.

A common clonal origin indicates intrapulmonary multifocal metastases in almost two-thirds of cases, while 36% of multifocal NSCLC display unique molecular profiles, which suggests separate primary tumors. Divergent KRAS and/or EGFR mutations have been observed in 8% of cases [6]. The same research studied the clonal relationship of multifocal NSCLC with indistinguishable histomorphology in 78 patients by polymorphic short tandem repeated markers and mutation testing of KRAS and EGFR [6]. This could provide remarkably increased response rates and better treatment outcomes, compared to ordinary histopathology-based stratification. This increased response rate is already the case with tyrosine kinase inhibitors (TKIs) and ALK inhibitors.

### 1.7. Histology

Diagnosis of lung cancer is challenging. Resected tumors provide histological tissue, and diagnosis can almost always be obtained. However, there are a lot of situations where obtaining adequate material for diagnosis is challenging in initial biopsies, and a lot of tumors are not operated on at all. An additional challenge is presented by known intratumor heterogeneity, which must be considered, especially when histological material is limited and not representative of the entire tumor. However, there can be small lesions or a situation that does not require an operation. In those clinical cases with small lesions requiring biopsies, histological tumor sampling remains difficult, and obtaining biopsy samples for thorough pathological assessments is difficult. Often, molecular pathology is simply not done. In some cases, only cytology is available, and further sampling is not possible because of the lesion location or the patient's low lung function. Treatment will begin, based on a fine-needle biopsy, or even a sputum sample, but there must be evidence of cancer. At the very least, lesions should behave like lung cancer. Different lung cancer types and different NSCLC cell clones behave differently and require different treatments.

There must be clinical confirmation of cancer, since oncology treatments generate so many side effects that clinical indication is required for their use. For a proper diagnosis, adequate histological or cytological material is required for morphological assessments, immunohistochemistry, and gene testing in cases of adenocancer. Here rebiopsy means biopsy after cancer progression on initial therapy and its role will be comprehensively summarized and broadly discussed in lung cancer.

## 2. Materials and Methods

This review is based on a PubMed search for the terms* rebiopsy* and* lung cancer* (Table 3). Publications in languages other than English and trials involving non-human subjects were excluded. Fourteen publications were reviewed, and a classification was performed with the predetermined variables listed in Table 3. The number of publications and trial protocols cited are as researched in March 2014. Additionally,* recurrent lung cancer* and* relapsed lung cancer* search terms were used resulting in 5225 and 1182 hits, but no additional articles were found by combining them with a* rebiopsy* search term. In order to check other articles and validate the search procedure, a repeated search was performed with the terms* repeated biopsy*,* lung cancer*, and* clinical*. It produced 544 hits from year 1975 to date. All abstracts were reviewed, and adequate articles that focused on rebiopsy were selected and included in this literature review. Two articles and two letters to the editor were evaluated for additional adequate information, and were subsequently incorporated into the analysis as additional articles.

## 3. Results

A PubMed search of the term* cancer diagnosis* produced almost 2 million hits. With the term* clinical biopsy*, there were 152,197 hits. This number dramatically decreased when the search was conducted for both* cancer diagnosis* and* clinical biopsy* or with lung cancer terms (see Table 3). Combining the term* rebiopsy* with* colon cancer*,* lung cancer*,* breast cancer*, and* prostate cancer* produced two, 16, 23, and 14 hits, respectively (Table 3). Of the articles with all indications, abstract analysis revealed that DNA and mutations were central to 12 and 11 articles, respectively, while histology was discussed in 235 of 309 articles with the term* rebiopsy*. No review articles were found in the area of rebiopsy in lung cancer.

Eighteen articles with the search terms* rebiopsy* and* lung cancer* were targeted for further analysis (Table 3). These articles were used to find more suitable works, which were then referenced. Four articles dealt with other cancers and were excluded from further analysis. The remaining 14 articles focused on NSCLC (Table 4). Details of major findings are given for each article. Four were case reports. One was about the pharmacoeconomic aspects of rebiopsy, and ten were original articles. Of these ten articles, one was a prospective clinical trial report, and one reported extensive mutation genotyping. Two articles focused on a specific gene expression, while the remaining six focused on tyrosine kinase (TK) resistance and mainly discussed the most frequent secondary mutation T790M.

### 3.1. Chemotherapy and Gene Expression

The prospective study assessed if chemotherapy selection based on* in situ* excision repair cross-completion group 1 (ERCC1) and ribonucleotide reductase M1 (RRM1) protein levels would improve survival in patients with advanced NSCLC [7]. A total of 275 eligible patients were randomly assigned to the control arm with gemcitabine/carboplatin or the trial's experimental arm. Chemotherapy therapy was given based on protein levels at repeated biopsy: if RRM1 and ERCC1 were low, gemcitabine/carboplatin were given; if RRM1 was high and ERCC1 was low then docetaxel/carboplatin were given; if RRM1 was low and ERCC1 was high then gemcitabine/docetaxel were given; if both were high then docetaxel/vinorelbine were given. While no statistically significant differences were observed between the experimental and control arms in PFS (progression free survival) (6.1 months versus 6.9 months) or overall survival (11 months versus 11.3 months), a subset analysis revealed that patients with low levels of both proteins who received the same treatment in both treatment arms had a statistically better PFS (*P* = 0.02) in the control arm (8.1 months) than in the experimental arm (five months). This study was in newly diagnosed patients with advanced-stage NSCLC. However, a repeated tumor biopsy without complications was needed in 17% of cases to ensure enough material for protein-level measurements [7]. This study gives a prospective setup for repeated biopsies, and even in the chemotherapy context may warrant conducting proper justification and direct chemotherapy.

Jakobsen et al. published two studies about specific gene expressions at the protein level obtained using immunohistochemistry. They discovered that thymidylate synthase (TS), which was a potential predictive marker for treatment efficacy with pemetrexed, did not significantly change in rebiopsied lung tumors compared to primary tumors in 65 NSCLC patients taking after preoperative carboplatin and paclitaxel [8]. In another study, 65 NSCLC patients taking preoperative carboplatin and paclitaxel and a group of 53 NSCLC patients treated with surgery alone showed no statistically significant change between primary and rebiopsy material of lung tumors in class-III-beta-tubulin expression, which may be a potential predictive factor for microtubule interfering cytotoxic drug treatment [9]. In these situations, the biomarker was not valid and thus rebiopsies were not justified. However, there was intratumoral heterogeneity in both studies, which highlighted the need for sufficient representative material for diagnosis.

### 3.2. Tyrosine Kinase Inhibitors and Resistance

Understandably, the main area for rebiopsies is among TKIs in adenocancers of NSCLC. All patients with EGFR-mutant lung cancers eventually develop acquired resistance to EGFR TKIs. This is associated with second-site mutations in the EGFR kinase gene (e.g., T790M), amplification of alternative kinases (e.g., mesenchymal-epithelial transition factor, MET), histologic transformation to small cell lung cancer (SCLC), and epithelial to mesenchymal transition. Various mechanisms have been identified to account for resistance, and many methods have been proposed to overcome resistance, especially caused by T790M [4, 10, 11]. The EGFR mutation T790M is reported in approximately half of adenocancers with acquired resistance to EGFR inhibitors and is a potential prognostic, predictive biomarker. Patients with EGFR-mutant lung adenocarcinoma develop acquired resistance to EGFR TKIs after a median of 10 to 16 months. In half of these cases, a second EGFR mutation, T790M, underlies acquired resistance. However, rebiopsy to confirm T790M status can be challenging due to limited tissue availability and procedural feasibility. Furthermore, little is known of the differences among patients with or without T790M mutation. Here, various rebiopsy studies reporting the frequency of T790M, reporting analysis for EGFR/ALK mutations and reporting responses to EGFR TKI are described. When there is a mechanism of resistance found, that is potentially actionable, new drug development could be initiated. So that for T790 mutations found, a T790M mutant specific inhibitors could be developed and, for MET amplification, a MET inhibitor could be tested.

A mutation genotype was investigated in a large, 155-patient study reported by Yu et al. [12]. Adequate tumor samples from rebiopsies for molecular analysis were obtained in lung adenocarcinoma tumors with acquired resistance to erlotinib or gefitinib. Sample material included fine-needle aspirations, core biopsies, surgical samples, and cytology from malignant effusions. There was one recorded complication of pneumothorax requiring a catheter placement. Furthermore, sites of rebiopsies included lung tumor (82), pleural effusions (14), bone (9), liver (13), lymph nodes (9), peritoneal fluid (1) and central nervous fluid (1), and other organs (9). The tissue samples were obtained via operational procedures in 17 cases, including 10 brain resections, 5 lymph node excisions and 3 adrenalectomies and two autopsies. Of these 155 patients, 98 had second-site EGFR T790M mutations (63%; 95% confidence interval [CI], 55%–70%). Four samples had small cell transformation. MET amplification was seen in four of 75 samples, and HER2 amplification was seen in three of 24 samples. No acquired mutations were observed in PIK3CA, AKT1, BRAF, HER2, KRAS, MEK1, or NRAS genes (0 of 88). The study identified EGFR T790M as the most common mechanism of acquired resistance, whereas MET amplification, HER2 amplification, and small cell histologic transformation occurred less frequently. The authors concluded that more rebiopsy studies were needed to characterize molecular alterations in situations of acquired resistance to EGFR TKIs [12].

Using a highly sensitive, locked nucleic-acid (LNA) PCR/sequencing assay with an analytical sensitivity of approximately 0.1%, T790M was detected in as many as 68% of patients with acquired resistance presenting either relapses or metastases. Tumor samples (153 samples in 121 patients) included the samples from clinically required procedures in 84 cases (e.g., 11 VATS biopsies, 6 lung resections, 3 image guided lung biopsies, and 2 fine-needle biopsies and 26 pleural effusions). In addition, the samples were obtained from other organs than lung in resections (14), biopsies (12), and fluid aspirations (8). The samples were studied for sensitizing EGFR mutations [13]. A total of 121 patients were rebiopsied and samples underwent tissue sampling. Of these, 104 (86%) samples were successfully analyzed for sensitizing EGFR mutations. Most failures were related to low tumor cell content. All patients (61) with matched pretreatment and resistance specimens showed susceptibility to the original sensitizing EGFR mutation. Standard T790M mutation analysis of 99 patients detected 51 (51%) mutations. Retesting of 30 EGFR-negative patients by the LNA-based method detected 11 additional mutations, for an estimated prevalence of 68%. MET was amplified in 11% of cases (4/37). The authors concluded that rebiopsy of lung cancer patients with acquired resistance was feasible and provided sufficient material for mutation analysis in most patients [13].

Of 126 patients referred for rebiopsy with NSCLC that was resistant to conventional chemotherapy or EGFR TKIs, 94 patients were selected for rebiopsy [14]. CT chest images excluded 32 patients. Percutaneous transthoracic lung biopsy was performed with a CT-guided, C-arm cone-beam, which had a technical success rate of 100%. In 75 (80%) of the 94 patients, specimens were adequate for mutational analysis. Thirty-five specimens were tested for EGFR mutation, 34 for ALK rearrangement, and six for both. The results were positive for EGFR-sensitizing mutation (exon 19 or 21) in 20 patients, EGFR T790M mutation in five, and ALK rearrangement in 11. Rebiopsy complications occurred in 13 (14%) patients. The study concluded that rebiopsies are feasible and safe when applying rigorous CT criteria and provide adequate material for gene analysis [14].

A study of 93 patients with EGFR-mutant lung cancer and acquired resistance to EGFR TKIs compared T790M status in terms of postprogression survival and characteristics of disease progression [15]. Mutation of T790M was observed in the initial rebiopsy specimens from 58 patients (62%, 95% CI: 52–72). T790M was more common in biopsies of lung/pleura tissue and lymph nodes than in other sites and it was more likely to progress in an existing site of disease than in new sites. Patients with T790M had a significantly longer postprogression survival time than patients without. Additionally, patients without T790M more often progressed to tumors in new, uninvolved organs and had a poorer performance status at time of progression. This study suggested that T790M serves a prognostic value that can be found by rebiopsy. Among patients with acquired resistance to EGFR TKIs, the presence of T790M defines a clinical subset with a relatively favorable prognosis and slower progression. The authors concluded that knowing T790M status was essential for clinical treatment decision making and understanding results of clinical trials after TKI use [15].

A study investigated 78 EGFR-mutant patients who underwent rebiopsy after TKI failure [16]. A sensitive, peptide nucleic acid-LNA polymerase chain-reaction clamp method was used in EGFR mutational analyses. The study found that patients with T790M after TKI failure had better prognoses than those without T790M. The T790M mutation was only identified rarely in four (17%) of 24 central nervous-system lesions and 22 (41%) of 54 other lesions (*P* = 0.0417). Median PFS was 31.4 months in 26 patients with T790M, and 11.4 months in 52 patients without T790M (*P* = 0.0017). In the multivariate analysis, statistically significant factors for longer PFS included positive for T790M, good performance status, and no carcinomatous meningitis [16].

Postprogression tumor specimens were prospectively collected for T790M mutation analysis in 70 NSCLC patients with acquired resistance to initial EGFR TKIs [17]. Thirty-six patients (51%) had T790M mutation in the rebiopsy specimen. There was no difference between the pattern of disease progression, PFS for initial TKIs (12.8 and 11.3 months), post-progression survival (14.7 and 14.1 months), or overall survival (43.5 and 36.8 months) in patients with and without T790M. After rebiopsy, 34 patients received afatinib treatment. The response rate was 18%, and the median PFS with afatinib was 3.7 months for the entire group and 3.2 and 4.6 months, respectively, for the subgroups with and without T790M. This means that there might be benefits for directing subsequent TKI therapies according to T790M status. Although T790M had no prognostic or predictive role in this study, identifying T790M as an acquired resistance mechanism was clinically feasible. Further research was felt to be necessary to identify patients with T790M-mutant tumors who might benefit from new T790M-specific TKIs currently in development [17].

### 3.3. Pharmacoeconomic Study

One report evaluated rebiopsy in NSCLC by cost-benefit modeling [18]. A decision-analysis model compared the costs and effects of platinum combination chemotherapy (carboplatin and paclitaxel; carboplatin and pemetrexed; and carboplatin, pemetrexed, and bevacizumab) with erlotinib therapy in patients with EGFR mutation-positive tumors. Compared with a combined carboplatin paclitaxel regimen, targeted therapy based on testing available tissue yielded an incremental cost-effectiveness ratio (ICER) of $110,644 per quality-adjusted life year (QALY). The rebiopsy strategy yielded an ICER of $122,219 per QALY. With a willingness to pay of $100,000 per QALY, the testing strategy was cost-effective 58% of the time, and the rebiopsy strategy was cost-effective 54% of the time. Compared with carboplatin, pemetrexed, and bevacizumab, ICERs were $25,547 per QALY for the testing strategy and $44,036 per QALY for the rebiopsy strategy. Personalized therapy with an EGFR-TKI was more favorable when the nontargeted chemotherapy regimen was more expensive. The authors concluded that cost-effectiveness analysis supports testing for EGFR mutations in patients with Stage IV or recurrent lung adenocarcinomas, performing rebiopsy if insufficient tissue is available for testing and treating patients with EGFR mutations with erlotinib as a first-line therapy. However, this study assumed that erlotinib offered a PFS benefit, and total costs greatly depended on costs of nontargeted chemotherapy, which could also depend on the health care system. QALY costs were much higher in the erlotinib group, and rebiopsy increased costs. In practice, patients tend to receive both targeted therapy and chemotherapy as the cancer evolves, so crossover is evident, and it is difficult to extract a single therapy element.

### 3.4. Case Reports

Four case reports were identified. Two of the reports dealt with rebiopsies on cancer progression and two additional ones were about insufficient initial biopsy and the necessity to perform repeated biopsy to obtain sufficient material for a proper diagnosis. The first case highlighted acquired EGFR-TKI resistance through transformation to the high-grade neuroendocrine carcinoma spectrum and that such transformation might not be evident at time of progression on TKI therapy [21]. A case of relapsed, EGFR exon-19 deletion, lung adenocarcinoma was treated with erlotinib and cisplatin-pemetrexed after resistance. Liver rebiopsy on progression identified an afatinib-resistant cancer with combined SCLC and NSCLC within neuroendocrine morphology, retaining the EGFR exon-19 deletion. Several acquired resistance mechanisms of EGFR-mutant lung adenocarcinoma to EGFR-TKI therapy were described, the most recent being transformation to SCLC [21].

The second case report demonstrated repeated responses to EGFR TKIs in a woman with adenocarcinoma and no history of smoking [20]. After six cycles of gemcitabine and cisplatin, the patient was treated by gefitinib for four months until progression. Following six cycles of third-line pemetrexed, gefitinib retreatment was initiated, with partial response for six months. After progression, the patient was recruited for an irreversible EGFR inhibitor trial. Time to progression was 11 months. Although EGFR direct sequencing on the initial diagnostic specimen revealed a wild type (nonmutated), rebiopsy of a progressed subcarinal node was performed at the end of the trial. Analysis showed an EGFR of mutation of L858R/L861Q [20].

The third study addressed the problem of tumor heterogeneity encountered in small bronchoscopic biopsies and the difficulties of evaluating the histological subtype in poorly differentiated carcinomas [19]. Initial diagnosis of squamous cell cancer (SCC) of the lung obtained by bronchoscopic biopsy was based on immunohistochemical staining only by positive results for cytokeratin (CK) 5/6 and p63 because morphological diagnosis was not possible. However, bronchoscopic repeated biopsy showed a mixed squamous/glandular immunophenotype with nests of undifferentiated tumor cells. There was weak immunoreactivity of some tumor cells for CK5/6 and p63 and no positivity of some tumor cells for thyroid transcription factor-1. In addition, an EGFR mutation was found in exon 21 (L858R). This was missed on initial biopsy. The patient achieved TKI and prolonged clinical benefit from treatment. The authors concluded that initial bronchoscopy should be performed by an experienced pulmonologist to obtain sufficient material from different areas of the tumor. In the era of targeted therapy, a patient having a history of remote smoking in cases of not-otherwise-specified (NOS) NSCLC that favors SCC should also provoke EGFR mutation testing [19]. Similarly, the fourth study also addressed the importance of adequate material for pathological evaluation in a report of five cases of regenerative, atypical squamous metaplasia at the site of a previous bronchial biopsy that was unnecessarily resected based on erroneous diagnosis of squamous cell carcinoma on repeated biopsy [22].

### 3.5. Additional Articles

In order to check for other articles and validate the search procedure, the search terms* repeated biopsy*,* lung cancer*, and* clinical* were entered, generating 544 hits. All abstracts were reviewed, and four additional articles were selected for this review: one case report about rebiopsy and three others dealing with repeated biopsy: two original articles and one letter to the editor.

A case report in a letter discussed an 80-year-old male with relapsed EGFR exon-19 deletion lung adenocarcinoma treated with EGFR-TKI. There were poor response and rapid increase of serum neuron-specific enolase [23]. Rebiopsy characterized transformation from NSCLS adenocancer to SCLC, and the EGFR mutation remained.

Three additional articles were about repeated biopsy rather than rebiopsy. Welker et al. [24] studied 118 patients with a solitary lung nodule (4 cm or smaller) who underwent transbronchial biopsy, percutaneous needle aspiration, clinical observation, repeat CT scans, and repeated biopsies. The mean follow-up was four years. The incidence of malignancy was 61%, and the positive predictive value, negative predictive value, sensitivity, specificity, and accuracy were all 100%. Moreover, this procedure reduced the incidence of unnecessary surgical excision of benign nodules from 60% to 5% [24]. Another letter to the editor stated that repeated needle biopsies were recognized to be safe and accurate in the management of a solitary pulmonary nodule [25]. The second original article was a retrospective study of 836 cases. Ninety-five cases with fine-needle aspiration +/− core biopsies over a five-year period were identified initially as nonmalignant [26]. Of these, 21 were confirmed later benign, and the remaining 74 included 53 initially benign and 21 nondiagnostic specimens. Seven of the 53 benign (13%) and six of 21 nondiagnostic specimens (29%) were malignant at excisional biopsy during radiologic follow-up. Sixteen of 95 cases (17%) had postprocedural pneumothorax that required a chest tube [26]. Therefore, repeated biopsy or resection is necessary for benign nonspecific and nondiagnostic biopsy results due to an unacceptably high rate of malignancy.

### 3.6. Safety

Serious complications in rebiopsy are rare. As there is already an initial diagnosis available, additional biopsies are carefully considered. Patients with lung cancer tend to develop metastases and especially liver and lymph node lesions are highly accessible for a biopsy. Probably a selection of biopsy sites has impact on low number of reported complications. One serious complication among 155 rebiopsies patients (12) and 13 minor complications in 94 patients (14) were reported in articles of this review. Additionally, no complications in 47 biopsied patients were reported by Bepler et al. in their repeated biopsy article [7]. In conclusion, rebiopsy appears to be safe when biopsy sites are carefully selected and the risk evaluation is made before rebiopsy.

## 4. Discussion

PubMed results reflect a lack of activity in rebiopsy for many indications, such as colon and lung cancers. Only 14 articles were found about rebiopsy in lung cancer by the search terms* rebiopsy *and* lung cancer* (Table 4). Prostate cancer had more hits (104) on the term* rebiopsy*. This reflects the attitude among urologists of actively performing repeated biopsies in follow-up and rebiopsies on relapses on prostate cancer patients. Of course, in the first place, it needs to recognize that the multiple biopsies are easier to do in prostate cancer than in lung cancer because of anatomical accessibility, lesion location, and minimal risk of complications. The situation with breast cancer is similar. The location of tumor relapse in breast tissue is usually accessible, but enlarged lymph nodes may be situated in places where performing a rebiopsy would pose too great a risk.

Solid tumors have a heterogeneous histological background, which makes it impossible to cover all metastases, even with only one highly targeted agent, which can only block one-cell clone at a time. In tumor growth and spread, cancer clones are probably randomly selected to survive, some of which may be resistant to given therapies, having an edge over other cell clones [27]. Furthermore, metastasizing involves one cell type and originates from one cell clone. New therapies block certain cell clones but miss others that develop based on other mutations [28, 29]. Therefore therapy fails, and redirection is needed.

In the optimal situation, therapeutic effect should be constantly monitored by repeating the histological examination, as the primary tumor can change. One clone or two clones may become resistant to a given therapy and dominate. In the metastasizing process, a limited number of cells fix themselves on remote places in the body. Some of these cells can avoid immunoreaction and start forming metastases. So a metastasis of a solid tumor can be very different from its parent tumor. Rebiopsy of lung cancer patients with acquired resistance is feasible and could provide sufficient material for mutation analysis in most patients [13]. Using a highly sensitivity method, a LNA PCR/sequencing assay, T790M, was detected in up to 68% of these patients, which was 12% more than with ordinary analysis methods.

Rebiopsies are widely used in cancers other than those in the lung. In prostate cancer, repeated biopsies and rebiopsies are readily performed, when prostate specific antigen (PSA) is increased, because doing so is easy, as there are no vital organs in the neighborhood of the prostate [30]. Similarly, rebiopsies are often performed in breast cancer to confirm cancer relapse and provide characteristics of a new breast cancer lesion. This will direct treatments, such as hormonal treatment in hormone receptor-positive cases. It will also confirm if the mutation in the HER2 oncogene and the elevated levels of HER2 protein are present, which triggers use of targeted therapies [31, 32]. It is difficult to access bone lesions and to retrieve good histological samples, and consequently bone lesions are normally not biopsied. The metastatic lesions were rebiopsied by core needle aspiration, or CT- or ultrasound-guided biopsy with no major complications. Additionally, rebiopsies may show a second malignancy [32, 33].

In neuroendocrine lung cancers, rebiopsy is widely used to pick up transformations to more aggressive types of cancer, such as small cell cancers. Transformation is also highly important to uncover in cases of suspected lymphoma relapse, for example, in thoracic area. There is also increased risk of secondary cancer in areas that have been radiated in Hodgkin's disease. The risk increases remarkably after decades from given radiotherapy. In certain cases, rebiopsy is not recommended. Schneider et al. [34] recommended omitting rebiopsy from clinical practice in esophageal cancer for objective response evaluation, based on his prospective study of 80 patients [34].  Table 5 summarizes the general reasons for not performing rebiopsy. The common reasons for not doing rebiopsy are that it is not routine practice, the anatomical location for the target tumor may make the operation too risky, and general perceptions that there is high risk involved.

One clear benefit from rebiopsy in treatment of NSCLC is that it provides an updated look at tumor characteristics, which can be used to redirect treatments [4]. This was demonstrated in the case reports addressing individualized approaches to lung cancer treatment. There was tumor heterogeneity in small bronchoscopic biopsies and challenges in histological subtyping of poorly differentiated carcinomas, repeated responses to EGFR TKIs based on EGFR mutation (in spite of initial wild-type characterization), and acquired EGFR-TKI resistance through transformation to the high-grade neuroendocrine carcinoma spectrum. All these cases highlight a need for rebiopsy.

**Figure 1:**
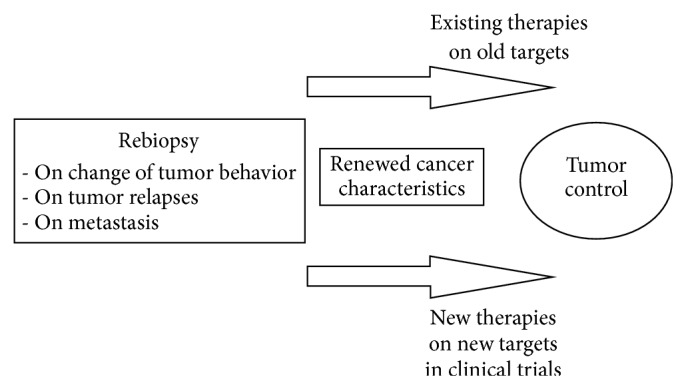
Role of rebiopsy in NSCLC treatment selection. Rebiopsy will renew tumor characteristics and give opportunity to act on changes of tumor behavior. Rebiopsy can confirm old existing targets when current therapies are allowed, or it can find new targets that need to be treated with new drug molecules in clinical trials. Thus changes in treatment facilitate better tumor control.

Figure 1 summarizes the potential benefits of rebiopsy in reassessing treatment options.  Table 5 lists main reasons not to perform rebiopsy, while Table 6 gives recommendation for rebiopsy in management of NSCLC. It can be important in treatment control when tumor behavior changes, as happens in a transformation into a more aggressive cancer type. It is important to get a look at changed tumor characteristics to determine the proper action. For example, neuroendocrine lung cancer can switch to SCLC type, which could be detected by rebiopsy. Tumor characteristics are important in directing treatments. Old targets can validate the choice to use existing and previous therapies. Moreover, material from rebiopsy makes it possible to explore a new target and to conduct clinical trials on new molecules [35]. When a TKI is used in NSCLC, there is a resistance tendency that becomes evident within two years. Some patients develop treatment resistance quicker than others, and rebiopsy is needed to confirm progression and look at new molecules that are being developed to overcome resistance. Without doing a rebiopsy to investigate the type of resistance and new targets, it would not be possible to use therapies against resistance in a controlled manner. One obstacle to drug development is that second-line patients with adequate tumor recharacterization to indicate gene alteration are difficult to find because rebiopsies are not customarily performed. However, nearly all clinical study protocols in relapsed adenocancer NSCLC now require a rebiopsy option to gather histological samples.

Novel immunotherapy strategy is pending on histological definition of targets in tumor, and those targets can change in time [36, 37]. So it is essential to search, for example, PD-L1 positivity for confirming reactivity of lung cancer on nivolumab before initiating treatment [38, 39]. PD-1 is expressed by activated T cells and down modulates T-cell effector functions on antigen-presenting cells; and in cancer patients, its expression on tumor-infiltrating lymphocytes and its interaction with the ligands on tumor and immune cells in the tumor microenvironment undermine antitumor immunity [40]. As PD-L1 measurement is done regularly in clinical trials that will be used in registration purposes, future treatment instructions after approval by drug agencies will include PD-L1 check before starting nivolumab. PD-L1 can be measured at protein level by immunohistochemistry, but there are only centralized measurements available at this early stage. This makes it necessary to send samples first for EGFR and ALK testing and then if negative send them to different central laboratory for PD-L1 testing, and less than 40% of samples will turn negative. This adds time for treatment decision-making and may slow down remarkably clinical trials. In addition, in case of relapses, patients cannot be entered into the trial if there are no rebiopsies done at relapse, as is usually the case. At the end, there will be no possibility to include patients in the treatment after the drug is approved by drug agencies, if a fresh rebiopsy is missing.

Rather than performing rebiopsies, some researchers have proposed analyzing serum for tumor DNA. Recent studies show that genomic alterations in solid cancers can be characterized by massively parallel sequencing of circulating, cell-free tumor DNA released from cancer cells into plasma. This represents a noninvasive liquid biopsy [38–40]. Cell-free DNA fragments from multiple lesions in the same individual all mix together in the peripheral blood. Therefore, serum tumor DNA is likely to contain a wider representation of the genomes from multiple metastatic sites, whereas a single biopsy may miss them [41]. Furthermore, intratumor heterogeneity in renal cell cancer makes it difficult to fully characterize primary tumor material and metastases that may be derived from a subclone missed in the primary tumor biopsy [42]. Similar situations were found in breast cancer [43] and probably in other solid cancers, including lung cancer [42]. When this new technology is clinically available, it will revolutionize NSCLC treatment with TKIs, as the development of resistance could be followed frequently and without rebiopsy restrictions, and further treatments could be properly redirected. Exceptions may occur where this approach may not work, as in immunotherapy. However, more research is needed, along with development of a methodology to suit clinical practice, which will certainly take many years. Meanwhile, it is important to use available methods in all clinical practices and to bridge new methodology with the old data, which will require rebiopsy material.

## 5. Conclusions

This review shows that rebiopsy is feasible in NSCLC, and success rates can be high if rebiopsy is accompanied by adequate evaluation before biopsy. As rebiopsy can be valuable method in clinical practice to help in selecting more efficient therapies for NSCLC patients, it should be performed more often (Table 6). However, before performing rebiopsy, adequate evaluation of risks for complications should be performed including anatomic and technical aspects of accessing tumor. A patient overall condition should also be taken in account. In situations where no possibility for active oncological interventions can be considered, rebiopsy is not indicated. Use of rebiopsy may resolve the difficulties in sampling bias and selecting preexisting or forming new drug-resistant clones. In cases where treatment was selected based on tissue characteristics that change, the treatment selection process must be repeated while considering new characteristics of the tumor. In the near future, rebiopsy will be used to predict therapeutic resistance and consequently redirect targeted therapies. Rebiopsy is done after the initial biopsy that provided the diagnosis. It is important to remember that metastases may behave differently, and have remarkable differences in histology content. Primary tumors can develop in such a way that the original histological content will change. This can be enhanced by efficient cancer therapy that usually influences nearly all cells. However, those cancer cells that do not die can develop into resistant clones. It would be critical to know when this development occurs. Even with the development of promising, new noninvasive methods for following cancer characteristics in serum samples, rebiopsy material will be urgently needed to identify and ensure those characteristics. Rebiopsies should be performed on lung lesions that were inadequately sampled by an initial biopsy when new metastatic lesions or relapses occur, in order to confirm the nature of the lesions and select the optimal targeted therapy.

Accordingly, some clinical practice guidelines already include this recommendation. For example, the ESMO 2012 guideline of advanced NSCLC states that obtaining adequate tissue material for histological diagnosis and molecular testing is important to individual treatment decisions and that rebiopsy at disease progression should be considered [44]. Clinical treatment will benefit from accurate histological diagnosis, and patients will be offered more focused therapies. Figure 1 addresses the importance of rebiopsy in NSCLC, in which treatment control can be received by recharacterization of tumor and selecting proper treatment on defined targets. If there is no tumor tissue available from a relapsed or progressed primary tumor, changed tumor behavior and cancer transformation are missed, and the molecularly guided stratification of patients into redirected treatments fails to happen.

## Figures and Tables

**Table 1:** Techniques for obtaining tissue.

Method	Nature of sample	Size	Suitable for
Sputum	Cytology	50 mg	Limited immunohistology
Bronchoscopy brushing	Cytology	50 mg	Limited immunohistology
Fine needle biopsy	Cytology	100 mg	Immunohistology and PCR
Core needle biopsy	Histology	200–400 mg	Plus gene mutation testing, FISH, and DNA tests
Resection	Histology	>1 g	Plus exome tests, large immunohistology panels, and RNA tests (−70°C)

**Table 2:** Information from rebiopsy.

Standard of care	Experimental
Histologic	Proteomics
Immunohistochemistry	RNAsequencing
Molecular information	Exome analysis
EGFR/KRAS/ALK	

**Table 3:** PubMed literature search for rebiopsy.

Rebiopsy	309
+ Colon cancer	2
+ Lung cancer	16
+ Breast cancer	23
+ Prostate cancer	104
Rebiopsy histology	235
Rebiopsy DNA	12
Rebiopsy mutations	11

**Table 4:** Rebiopsy and lung cancer.

	Number of articles	Number of patients	Content	Reference
Case reports	4	8		[17–20]
Pharmacoeconomic analysis	1			[16]
Original articles	9			
		53	TS expression	[9]
		65	Beta tubulin	[8]
		70	T790 mutation	[15]
		78	T790 mutation	[14]
		93	T790 mutation	[13]
		94	EGFR mutations ALK rearrangement	[12]
		121	T790 mutation	[11]
		155	Mutation genotyping	[10]
		331	ECCI and RRMI proteins	[7]

**Table 5:** Why rebiopsy is not done in NSCLC.

(i) Not part of clinical routine	
(ii) Anatomical location is difficult for biopsy	
(iii) Sense of risk involved in rebiopsy	
(iv) Limited number of drugs that can be directed by rebiopsy	
(v) Only a few reports available in the literature	

**Table 6:** Recommendation for rebiopsy in NSCLC.

*When rebiopsy should not be performed*:	
(i) Too difficult a location for safe biopsy	
(ii) Result will not change treatment	
*When rebiopsy should be performed*:	
(i) If the prior specimen is too small for adequate tumor characterization, including genetic testing for predictive alterations	
(ii) If relapse happens a long time (six months) after CR treatment result	
(iii) If the new tumor behaves in a different way than expected from the primary tumor	
(iv) If new molecules entering clinical trials in the near future is foreseeable, such as adenocancer relapses	

## Abbreviations

CT: Computer tomography
PET: Positron emission tomography
VATS: Video-assisted tomography
EBUS: Endobronchial untrasound
EMN: Electromagnetic navigation
NSCLC: Non-small cell lung cancer
DNA: Deoxyribonucleic acid
RNA: Ribonucleic acid
EGFR: Epidermal growth factor receptor
ALK: Anaplastic lymphoma kinase gene
TP53: Tumor protein p53
KRAS: Kirsten rat sarcoma viral oncogen homolog
STK1: Serological thymidine kinase 1
TKI: Tyrosine kinase inhibitor
PubMed: An Internet site for biomedical literature
TK: Tyrosine kinase
T790M: A gatekeeper mutation in EGFR
ERCCI: DNA excision repair protein
RPMI: Disease resistance protein
PFS: Progression free survival
TS: Thymidylate synthetase
SCLC: Small cell lung cancer
MET: Mesenchymal-epithelial transition factor
PIK3CA: Phosphatidylinositol-4,5-biphosphate 3-kinase, catalytic subunit alpha
AKT1: RAC-alpha serine/threonine-protein kinase
BRAF: v-Raf murine sarcoma viral oncogene homolog B
NRAS: Neuroblastoma RAS viral oncogene homolog
HER2: Human epidermal growth factor receptor 2
LNA: Locked nucleic acid
PCR: Polymerase chain reaction
QALY: Quality adjusted life year
ICER: Oncremental cost-effectiveness ratio
CK: Cytokeratin
NOS: Not otherwise specified
PSA: Prostate specific antigen
ER: Estrogen receptor
PR: Progesterone receptor
PD-L1: Programmed death-ligand 1
T-cell: Lymphocytes maturing in thymus
ESMO: European Society of Medical Oncology.
